# Viscoelastic Liquid Matrix with Faster Bulk Relaxation Time Reinforces the Cell Cycle Arrest Induction of the Breast Cancer Cells via Oxidative Stress

**DOI:** 10.3390/ijms232314637

**Published:** 2022-11-24

**Authors:** Mazaya Najmina, Mitsuhiro Ebara, Takahito Ohmura, Koichiro Uto

**Affiliations:** 1 Research Center for Functional Materials, National Institute for Materials Science, 1-1 Namiki, Tsukuba 305-0044, Japan; 2 Graduate School of Science and Engineering, University of Tsukuba, 1-1 Tennodai, Tsukuba 305-8577, Japan; 3 Graduate School of Industrial Science, Tokyo University of Science, 1-3 Kagurazaka, Shinjuku, Tokyo 162-8601, Japan; 4 Research Center for Structural Materials, National Institute for Materials Science, 1-2-1 Sengen, Tsukuba 305-0047, Japan

**Keywords:** material-induced senescence, stress relaxation, breast cancer, multicellular aggregates, oxidative stress

## Abstract

The reactivating of disseminated dormant breast cancer cells in a soft viscoelastic matrix is mostly correlated with metastasis. Metastasis occurs due to rapid stress relaxation owing to matrix remodeling. Here, we demonstrate the possibility of promoting the permanent cell cycle arrest of breast cancer cells on a viscoelastic liquid substrate. By controlling the molecular weight of the hydrophobic molten polymer, poly(ε-caprolactone-*co*-D,L-lactide) within 35–63 g/mol, this study highlights that MCF7 cells can sense a 1000 times narrower relaxation time range (80–290 ms) compared to other studies by using a crosslinked hydrogel system. We propose that the rapid bulk relaxation response of the substrate promotes more reactive oxygen species generation in the formed semi-3D multicellular aggregates of breast cancer cells. Our finding sheds light on the potential role of bulk stress relaxation in a viscous-dominant viscoelastic matrix in controlling the cell cycle arrest depth of breast cancer cells.

## 1. Introduction

Although most viscoelastic matrices within the body tissues function as viscoelastic solids, some cellular processes and diseases also occur in the viscoelastic liquid matrix. Breast cancer affects 7.8 million women globally; the rate of occurrence increases with age [1]. Due to the mucinous breast cancer type (tan α = ~2.9), a breast cancer cell (25–30 Pa.s) is more fluid-like than a normal breast cell (50 Pa.s). The breast cancer cells disseminate into the soft (viscous-dominant) tissue, such as bone marrow (37.5–125 mPa.s) and cerebrospinal fluid (1 mPa.s), presenting a potential interplay between the viscous component of the matrix and the breast cancer progression [2,3,4,5]. Moreover, the increasing composition of hyaluronic acid, a viscoelastic liquid component that makes up the extracellular matrix (ECM), characterizes the invasion of breast ductal carcinoma implying the positive correlation between matrix viscosity and breast cancer progression [6].

The relapse of cancer in other organs post-treatment of breast cancer is still not completely understood. The disseminated breast cancer cells residing in the soft tissue, such as bone marrow or brain, usually remain dormant [7]. The dormant cancer cells reactivate upon various triggers, such as inflammation, ECM remodeling, or hormonal imbalance [8,9]. A soft matrix represented by hyaluronic acid hydrogel (~0.4 kPa) drives the reversible dormancy of breast cancer cell clusters in brain tissue after metastasis [10]. Although the mechanism of the matrix influencing the dormancy of breast cancer cells remains relatively unknown, dormancy is certainly a ticking bomb. Therefore, shifting this metastable state into a more stable senescence state can help avoid the reactivation of dormant cancer cells. The induction of senescence in cancer cells can be a novel strategy to selectively kill senescent cancer cells by senolytic therapy and improve conventional cancer treatment [11,12]. In addition to the common methods of inducing senescence in cancer cells (e.g., oncogene-induced senescence, chemotherapies, radiotherapies), we propose material-induced senescence, in which the senescence of cancer cells can be triggered by modulating the viscous properties of materials [13,14].

The viscoelastic system possesses a stress relaxation behavior defined as the resistance towards the force relaxing over time owing to the presence of a viscous component [15]. Stress relaxation of the tissue represents the ability of ECM to be remodeled upon the force transmission of cells which further determines the stem cell differentiation lineage, epithelial cell morphogenesis, and migration of cancer cells [16,17,18]. A study utilizing the alginate hydrogel with varied covalent crosslinking densities revealed that stress-relaxed substrate enhances the spreading ability of human mesenchymal stem cells. Also, faster stress relaxed hydrogel (τ_1/2_) = 80 s) in both 2D and 3D cell culture environments enhances the proliferation of breast cancer cells (MCF-7 and MDA-MB-231) through phosphoinositide 3-kinases (PI3K)/Akt-p27Kip1 signaling axis in anchorage-independent cell growth [19,20]. In another study, interfacial stress relaxation regulates the adhesion and migration of epithelium and controls the maturation of epithelial cell aggregation [21]. The enhanced matrix remodeling indicates the increasing number of senescent cells [22]. In a molecular clutch model, when the relaxation time of the substrate is faster than the integrin “clutch” binding, the cell adhesion is consequently impeded [23,24]. Moreover, inhibition of integrin-linked kinase triggers the senescence of gastric and colorectal cancer cell lines [25,26]. Altogether, these studies imply that stress relaxation of a matrix can potentially be manipulated to promote the senescence of breast cancer cells.

In this study, we aim to induce the senescence of cancer cells by developing a viscoelastic liquid substrate. This hydrophobic molten copolymer substrate (poly(ε-caprolactone-*co*-D, L-lactide), P(CL-*co*-DLLA)) has been previously confirmed to induce cell cycle arrest in breast cancer cell line (MCF-7) and lung cancer cell line (NCIH322) [13,14]. However, the substrate properties and molecular mechanism underlying the senescence induction of both epithelial cancer cell lines upon growing those cells on a viscoelastic liquid substrate are lesser known. Additionally, we aim to highlight that bulk relaxation of a viscoelastic liquid matrix determines the magnitude of breast cancer cell senescence. From the biomaterials design point of view, another study has provided insight into controlling the dormancy state reactivation of breast cancer cells (MDA-MB-231) by tuning the RGDs ligand density of a polyethylene glycol-based hydrogel [27]. Our study aims to demonstrate that varying the bulk viscoelasticity of a substrate can induce some breast cancer cells into both reversible and irreversible dormancy that is retained even after the cell replating experiment through a series of biomolecular assessments of cell-substrate adhesion, proliferation, metabolic activity, and cell cycle arrest.

## 2. Results

### 2.1. The Molecular Weight-Dependent Rheology and Surface Properties of P(CL-co-DLLA) Substrates

Three types of P(CL-*co*-DLLA) copolymers with different molecular weights (Table S1) were used to prepare cell culture substrates. Using gel permeation chromatography (GPC) relative to the polystyrene standard, the weight-average molecular weights (M_w_) of these copolymers were estimated to be 35, 41, and 63 g/mol with a polydispersity index (PDI) of 1.6–1.9 respectively (Table S1). The monomer composition (CL: DLLA) of each copolymer was controlled to approximately 1.6, as determined by ^1^H-nuclear magnetic resonance (NMR) spectroscopy (Table S1). According to the differential scanning calorimetry measurement, each copolymers showed an endothermic peak below 33 °C without any exothermic peak, indicating no remarkable crystallization temperature (Figure S1), suggesting that these copolymers are amorphous and exist as a polymer melt below the body temperature owing to the presence of amorphous DLLA units.

**Figure 1:**
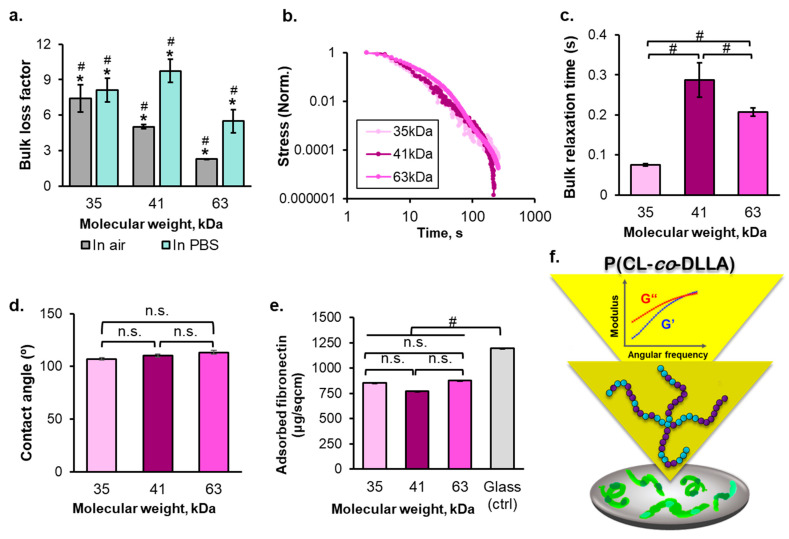
(**a**) Bulk loss factor of the copolymer substrates obtained by rheometer at 1 Hz angular frequency and 37 °C, (**b**) bulk stress relaxation of the copolymer substrates obtained by rheometer at 10% strain concentration (normalized to their each maximum individual stress), (**c**) bulk relaxation time of the copolymer substrate obtained by fitting the stress relaxation curve to the standard linear solid model, (**d**) the contact angle of the copolymer substrates, (**e**) the quantified amount of adsorbed fibronectin on each substrate, and (**f**) the schematic illustration of the fibronectin (green)-coated copolymer substrate used as a cell culture substrate. (Statistical analyses: Student’s *t*-test n.s. *p* > 0.05; # is the comparison between samples *p* < 0.05; * is the comparison between different treatment *p* < 0.05).

The frequency-dependent bulk rheological behavior of the molten copolymer at 37 °C showed that G″ initially exceeded the G′, followed by the appearance of G′′ and G′ intersection at a higher frequency, indicating the typical viscoelastic nature (Figure S2). The actomyosin contracts at a rate of 1 μm/s, and MCF-7 cells exert filopodia with ~1 μm [28,29]. Therefore, the G′ and G″ of the substrates were determined at the frequency of 1 Hz. At this frequency, the value of G′ was ~10% of that of the G″; thus, the bulk loss factor of these copolymers addresses the viscoelastic liquid nature of the copolymers (Figure 1a). The tendency of the bulk loss factor was inversely correlated to the molecular weight of the copolymer. In addition, the bulk loss factor measured in PBS suggests that the copolymers maintain their viscoelastic liquid nature under the cell culture condition. The purpose of varying the molecular weight of the copolymer was to obtain the bulk loss factor of 5–10; in this case, we particularly expected to have a copolymer with varied viscous components as the main design parameter. Altogether, our results showed that the rheology properties of these copolymers fulfilled our design expectations.

Next, stress relaxation is an important parameter to explain cell growth because it is responsible for the ability of cells to engage their clutch in transmitting their traction to enable spreading and actively deform their surrounding matrix for building a proper growth microenvironment [30,31]. The stress relaxation time obtained by fitting the individual normalized stress relaxation curve (Figure 1b) to the standard linear solid equation was hundreds of milliseconds. The cells respond to the force oscillation within the range of ~1 s, as reported by a previous study [28]. Therefore, the stress relaxation time of these copolymers was close to the matrix force sensing timescale of the cells. By controlling the molecular weight of the polymers, the stress relaxation halftime (τ_1/2_) of 80–290 ms was obtained (Figure 1c). Here, no linear correlation between the molecular weight and τ_1/2_ was observed (Figure S3). Because of the possibility of the PDI effect of the polymer, which a larger PDI results in a shorter relaxation time of the molten polymer, as shown in the previous experiments with polypropylene fiber [32]. Nanoindentation at 300 nm from a spin-coated substrate surface confirmed the negligible surface stress relaxation among those copolymers (Figure S3). The 10× higher surface τ_1/2_ obtained by nanoindentation compared with the bulk τ_1/2_ obtained by rheometer was due to the measurement temperature, that is, room temperature (25 °C). However, the melting temperature of the copolymers was higher than 25 °C (Figure S1). Consequently, the copolymer substrates were partially crystallized. The copolymers had a glass transition temperature (T_g_) around room temperature (Figure S1). As an amorphous polymer, the polymer chain mobility decreased below T_g_, as reflected by the drastic increase in the τ_1/2_. Other studies have reported that the surface stiffness of 5–200 nm from the surface of an amorphous polymer as the thin film was approximately 200% higher than its bulk stiffness under the mechanical contact loading following the increasing T_g_ to its glassy state. This is driven by the contact stress-induced formation of a confined phase, which is independent of the molecular weight and persistence length of the polymer [33]. This report was consistent with our data, where the surface τ_1/2_ of each polymer was invariant regardless of its molecular weight difference. In addition, the relaxation time of the polystyrene dish, which was used as a control to represent the solid surface, was 10^8^ ms. It was estimated based on polydisperse polystyrene, a type of polystyrene used to make commercial tissue culture dishes [34,35].

The cell culture substrates were prepared by spin-coating the three-copolymer solution on a coverslip glass resulting in ~20 μm thickness. After 1 d of culture, the thickness of the substrates decreased to ~15 μm (Figure S4). Despite the decrease in substrate thickness over 5 d of cell culturing, the substrate thickness remained in the acceptable range for avoiding the cell sensing of the underneath coverslip glass; because the MCF7 cell protrusion length was ~7 μm [36]. The interface of the substrates was relatively flat. Thus, negligible roughness difference among the substrates was observed (Figure S5). The contact angle also showed a negligible difference among the substrates (Figure 1d). All substrates were relatively hydrophobic (>90°), ensuring stability during the cell culture period until day 5. Moreover, the FRAP assay, which confirmed the lateral mobility of the substrates in PBS, demonstrated an insignificant change in substrate lateral mobility during days 1–5 of cell culturing (Supplementary Figure S6). The segmental mobility of spin-coated polymer film decreases as the surface roughness increases [37]. Therefore, the FRAP assay result implies that the surface roughness of all polymer substrates was not sufficiently altered after 5 d of cell culturing.

Before culturing the cells onto the interface of the substrates, fibronectin, an ECM protein, was coated onto the substrates to provide cell adhesion ligand. The micro–BCA assay was conducted to measure the concentration of unabsorbed protein, which was finally subtracted from the initial fibronectin coating concentration. The result suggests that the protein adsorption does not vary across the substrates, and the fibronectin adsorption onto the copolymer substrates was also lower than that of the control (coverslip glass) (Figure 1e). Despite the hydrophobicity of the polymeric substrates that would ensure protein adsorption [38], the viscoelastic liquid nature of the copolymers may inhibit the unfolding of fibronectin. The constant globular shape of an adsorbed protein on the viscoelastic liquid substrate results in comparatively lower protein adsorption compared to the control.

### 2.2. The Morphology of Multicellular Aggregates of Breast Cancer Cells in P(CL-co-DLLA) Substrates

**Figure 2:**
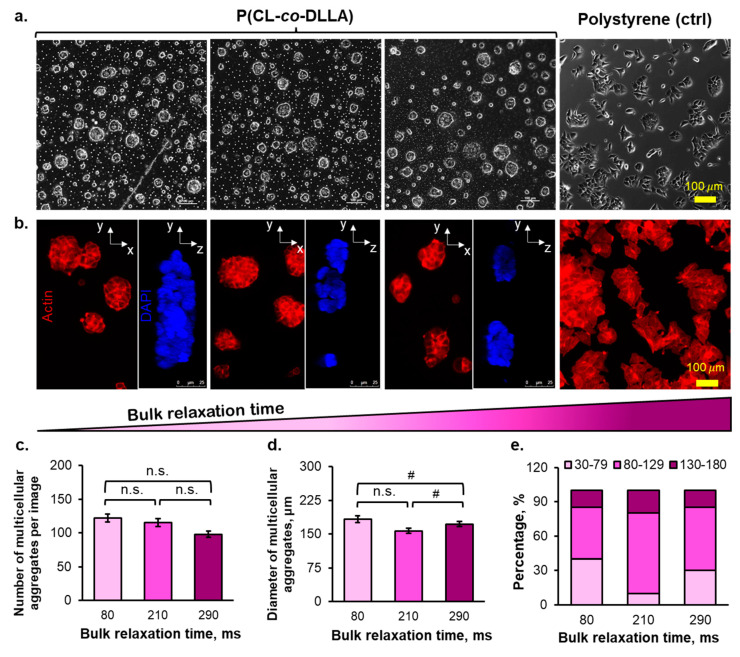
(**a**) The brightfield image (x–y) of the morphology of the multicellular aggregates of MCF-7 on day 5, (**b**) The actin (red) stained and z-axis view of DAPI (blue) stained- cells on day 5, (**c**) the number of generated multicellular aggregates on day 5, (**d**) the average diameter of the multicellular aggregates on day 5, and (**e**) the diameter distribution frequency of the multicellular aggregates on day 5. (Statistical analyses: Student’s *t*-test n.s. *p* > 0.05; # *p* < 0.05).

**Figure 3:**
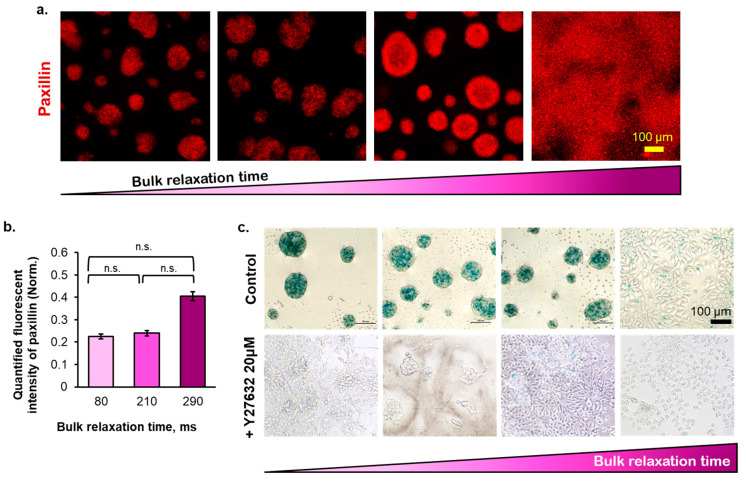
(**a**) The immunofluorescence-stained images of the cells on day 5 for paxillin, (**b**) the quantified expression of paxillin on day 5 (normalized to the paxillin expression in control substrate, polystyrene), and (**c**) the expression of chromogenic senescence marker (SA-β-Gal) with various condition (untreated, or treated with a ROCK inhibitor, Y27632). (Statistical analyses: Student’s *t*-test n.s. *p* > 0.05).

The MCF-7 cells instantaneously formed multicellular aggregates (Figure 2a,b and Figure S15a) 24 h after seeding onto the P(CL-*co*-DLLA) substrates regardless of the stress relaxation of the substrates, where the multicellular aggregates show the lack of actin fibers formation (Figure S15b). The cellular spreading event was commonly initiated by the ligand–integrin–actin linkage, followed by paxillin activation. Although the cells did not spread on the copolymer substrates, those multicellular aggregates were able to attach to the copolymer substrates as marked by the expression of paxillin, as a cellular attachment to the substrate can be marked by the recruitment of paxillin (Figure 3a,b, Figures S16 and S17). Both quantified immunofluorescent staining image and blot of paxillin (Figure S17a,b) indicate that 80–290 ms copolymer substrates promoted lower paxillin recruitment compared to the control substrate. The diameters of the multicellular aggregates increased gradually from days 1 to 3 of the cell culturing period and did not markedly increase from days 3–5 (Figure S7). However, there was an insignificant effect of bulk stress relaxation time of the substrates on the number and average diameter of the multicellular aggregates at day 5 of cell culturing (Figure 2c,d). The rapidly relaxed substrate induced the formation of multicellular aggregates with small diameters (<80 µm), whereas the diameters of the moderately relaxed substrate-formed multicellular aggregates were large (>130 µm) (Figure 2e).

Despite the negligible interfacial physicochemical properties among the substrates, the copolymer substrates did not form homogeneous multicellular aggregates. The number of small-sized multicellular aggregates was higher in the rapidly relaxed substrate than in moderately and slowly relaxed substrates (Figure 2e). This tendency was observed because a rapidly relaxed substrate enhanced the movement speed of the cells, thereby promoting more fusion of multicellular aggregates. Concerning the multicellular aggregate structure, the rapidly relaxed substrate formed a thicker structure in terms of multicellular aggregates’ depth, owing to the rapid fusion between small multicellular aggregates. The structure of the viscoelastic liquid–triggered multicellular aggregation was dome-like or semi-3D (Figure 2b and Figure S8). Altogether, the bulk stress relaxation time of the substrates only affected the multicellular aggregates diameter distribution of breast cancer cells over 5 d of cell culturing (Figure 2e).

### 2.3. The Correlation between Substrate Bulk Stress Relaxation and the Metabolic Activity, Proliferation, Viability, and Cell Cycle of the Breast Cancer Cells

**Figure 4:**
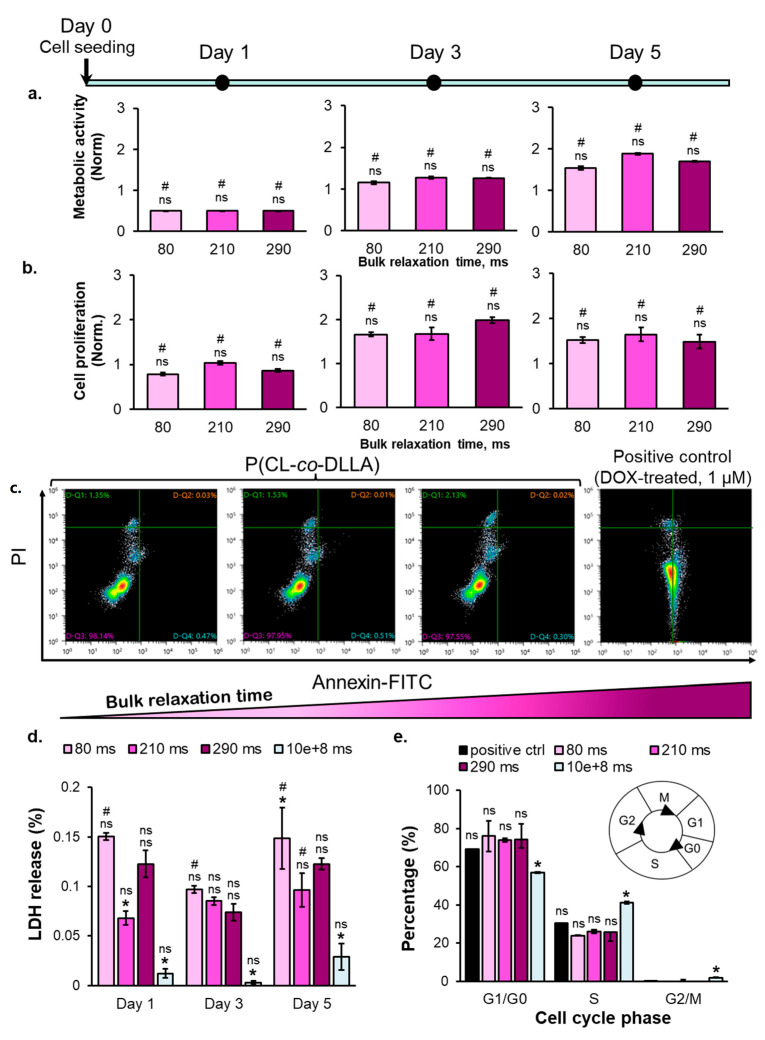
(**a**) The metabolic activity (NAD+ → NADH) of the cells on day 5, (**b**) the proliferation ability of the cells on day 5, (**c**) the expression of apoptosis & necrosis marker of the cells on day 5 on the substrates with various bulk relaxation time (from left to right: 80, 210, 290, and 10^8^ ms), (**d**) the percentage of cell death marker-LDH on day 5, and (**e**) the cell cycle distribution on substrates with various bulk relaxation time on day 5. (Statistical analyses: Student’s *t*-test ns *p* > 0.05; # is the comparison between each time point *p* < 0.05; * is the comparison between samples at the same time point *p* < 0.05).

The multicellular aggregates formed on the copolymer substrates did not show any substrate bulk stress relaxation time-dependent cell metabolic activity and proliferation ability on days 1 and 3 of cell culturing (Figure 4a). The cell metabolic activity on the copolymer substrates gradually increased from day 1 until day 5 (Figure 4a). Meanwhile, the proliferation ability of the multicellular aggregates on the copolymer substrates initially increased from days 1 to 3 and then did not increase anymore from days 3 to day 5 (Figure 4b). In contrast, the cells grown on the tissue culture plastic dish showed both increasing metabolic activity and cell number during the 5 d of cell culturing (Figure S9). The decreasing cell proliferation observed on the copolymer substrates on day 5 was due to the cell cycle arrest at the S phase (Figure 4e). Based on the cell cycle analysis (Figure 4e), most of the cells cultured on the copolymer substrates reside in the G0/G1 phase, and no cells were observed in the G2/M phase. The cells on the copolymer substrates on day 5 remained viable, as shown by the negative expression of the apoptosis marker (Annexin V) and necrosis marker (PI) in Figure 4c, along with the insignificant amount of lactate dehydrogenase (Figure 4d). On day 5, the metabolic activity, cell number, and percentage of cells in each cell cycle phase (except G2/M) show no correlation to the bulk relaxation time of the substrates. However, the percentage of S–phase–arrested breast cancer cells cultured on the viscoelastic liquid matrix was similar to the anticancer drug-induced senescent cells, regarded as the positive control in Figure 4e. Altogether, a threshold of substrate bulk relaxation time that can induce the cell cycle arrest of breast cancer cells on the P(CL-*co*-DLLA) substrate may exist under 80 and 210 ms. The multicellular aggregates on the copolymer substrates were stained positive for SA-β-Gal, a biomarker of senescence (Figure 3c, top panel). Therefore, the increase in metabolic activity, followed by the decrease in cell numbers, can be associated with senescence. Additionally, the inhibition of Rho-associated protein kinase (ROCK), a focal adhesion regulator, revealed that the senescence fate of the breast cancer cells on copolymer substrates greatly depended on the formation of multicellular aggregates. When the ROCK inhibitor was added, the senescence was inhibited, as indicated by the loss of SA-β-Gal (Figure 3c, bottom panel). Overall, all copolymer substrates with 80–290 ms bulk τ_1/2_ promoted the cell cycle arrest in the breast cancer cells after day 5 of cell culturing with a comparable level to the anticancer drug-induced senescence (positive control).

### 2.4. The Effect of Substrate Bulk Stress Relaxation on the Generation of Reactive Oxygen Species (ROS) in the Multicellular Aggregates of the Breast Cancer Cells

**Figure 5:**
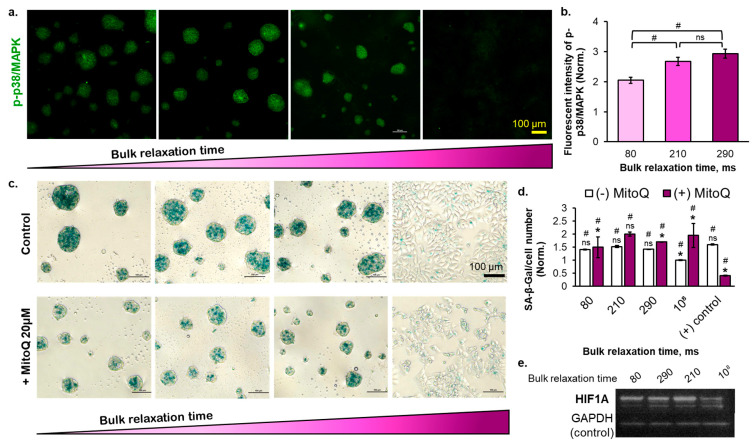
(**a**) The immunofluorescence staining images of the phosphorylated p38/MAPK on day 5, (**b**) the quantified expression of phosphorylated p38/MAPK based on its immunofluorescence images, (**c**) the expression of chromogenic senescence marker (SA-β-Gal) of untreated and ROS inhibitor (MitoQ) treated cells after day 5 of cell culture with scale bar= 100 µm, (**d**) the quantified SA-β-Gal before and after ROS inhibition with MitoQ, and (**e**) the western blot image of the HIF1A protein at day 5. (Statistical analyses: Student’s *t*-test ns *p* > 0.05; # is the comparison between drug-treated and untreated *p* < 0.05; * is the comparison between different samples *p* < 0.05).

ROS accumulation at a certain level triggers cell senescence. The cells spontaneously form multicellular aggregates on the copolymer substrates with semi-3D structures, thus potentially accumulating ROS. We hypothesized that the cells cultured on the copolymer substrates undergo cell cycle arrest due to the generation of ROS. After confirming the ROS generation through the 2′,7′-dichlorodihydrofluorescein diacetate (DCFDA) staining (Figure S10), the slowly relaxed substrate showed a higher amount of generated ROS. To verify this, we checked the p38/MAPK phosphorylation, which is known to be phosphorylated by ROS (Figure 5a,b). The result was consistent with the DCFDA expression result; the cells cultured on the slowly relaxed substrate demonstrated higher p38/MAPK phosphorylation. The multicellular aggregates formed at the interface of the substrate enlarged over the cell culture period (Supplementary Figure S5), leading us to hypothesize that the central region of the multicellular aggregates is a hypoxic environment. The HIF1A was intensely expressed on the copolymer substrates but dimly expressed on the polystyrene substrate (Figure 5e and Figure S12). This result was reasonable because the cells cultured on the polystyrene substrate were in a monolayer state. Nevertheless, the substrate bulk τ_1/2_ did not correlate with the HIF1A due to the negligible size difference observed among the multicellular aggregates on day 5 of cell culturing (Figure S7). Next, we treated the cells with MitoQ, a chemotherapy drug that increases ROS production in breast cancer cells to induce cell death [39]. The ROS level can determine the cellular fate (proliferation, apoptosis, senescence) in a dose-dependent manner [40]. By adding 20 µM MitoQ, the SA-β-Gal expression of the breast cancer cells cultured on all types of substrates increased (Figure 5d), implying that culturing the cells on a viscoelastic-liquid matrix increases the ROS level in cells sufficient to induce the early level of senescence. By adding MitoQ, the ROS level in cells increased senescence to a deeper level. In another study, the higher intensity of SA-β-Gal observed in the blood cells collected from aging humans correlated with the abundance of permanent senescent cells, which were marked by telomere dysfunction and impaired proliferation [41]. Therefore, the difference in SA-β-Gal intensity observed in our result, before and after MitoQ addition, implies the varied depth of senescence. In contrast, the MitoQ addition to a positive control (low DOX concentration-treated cells) caused a decrease in the SA-β-Gal expression (Figure 5d). Because of the possible synergistic anticancer effect between DOX and MitoQ, thereby increasing the ROS level sufficient to induce cell apoptosis with the MitoQ addition. Overall, ROS and the bulk τ_1/2_ of the substrate plays important roles in promoting the cell cycle arrest of breast cancer cells. Moreover, the threshold of substrate bulk relaxation time to induce the senescence of MCF-7 cells may lie between 80 and 210 ms, as indicated in Figure 5d (with MitoQ) and Figure 5b.

### 2.5. The Reversibility of the Breast Cancer Cells Senescence Fate Induced on the Copolymer Substrates

**Figure 6:**
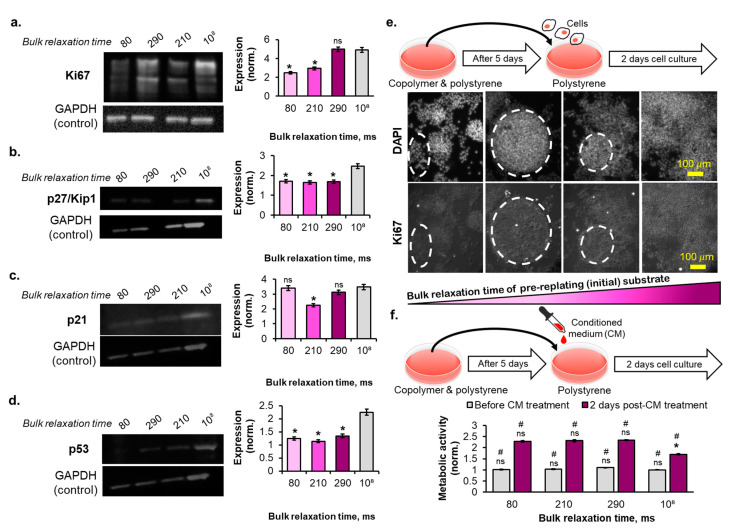
The western blot images of (**a**) Ki67, (**b**) p27/Kip1, (**c**) p21, and (**d**) p53 on day 5, and their quantified expression normalized to that of GAPDH, (**e**) the bulk $\tau_{1/2}$ of the copolymer substrates-dependent Ki67 expression level & DAPI staining images of the re-plated cells on the polystyrene dish after 2 days of cell culture, and (**f**) the metabolic activity of the cells after 2 days of cell culture on the polystyrene dish before and after supplementation with CM. (Statistical analyses: Student’s *t*-test ns *p* > 0.05; # is the comparison between untreated and CM-treated *p* < 0.05; * is the comparison between samples *p* < 0.05 [in (**a**–**d**), * refers to the comparison between each sample to 10^8^ ms substrate).

Finally, we elucidated the degree of cell cycle arrest in the breast cancer cells on the copolymer substrates on day 5 of cell culture. The GAPDH-normalized expression of the proliferation-related protein, Ki67, showed a positive correlation with the bulk τ_1/2_ of the substrates (Figure 6a and Figure S13). Meanwhile, the expression of the G1 phase cell cycle inhibitor protein, p27/Kip1, normalized to the GAPDH protein, demonstrated comparable and no correlation to the bulk stress relaxation time of the substrates (Figure 6b and Figure S13). In contrast, the expression of the S phase cell cycle inhibitor protein, p21, showed a biphasic correlation with the bulk stress relaxation time of the substrates (Figure 6c and Figure S13). Here, p21 was downstream of the p53 protein; thus, the p53 expression of the cells also showed a similar biphasic correlation with the bulk stress relaxation time of the copolymer substrates (Figure 6d and Figure S13). The p27/Kip1 protein expression level was relatively lower than that of p21. Western blotting results of p27/Kip1 and p21 were positively correlated with the cell cycle analysis result, where the cells cultured on the copolymer substrates were arrested during the S phase. p53 and p21 expression do not guarantee permanent or irreversible cell cycle arrest. Therefore, we performed the cell replating experiment by collecting the cells cultured in various substrates for 5 d and transferring them onto the polystyrene tissue culture dish. After 2 d of replating the cells into the polystyrene dish, certain numbers of cells cultured for 5 d on the viscoelastic liquid matrix did not regain the Ki67 expression (Figure 6e). The cells cultured for 5 d on a rapidly relaxed bulk τ_1/2_ showed a higher number of cell populations without Ki67 protein expression than those from the slowly relaxed bulk τ_1/2_ substrates (Figure 6e, immunofluorescent images). The cells that did not recover the Ki67 expression also did not show complete spreading behavior and formed an aggregated cell cluster instead (Figure 6e, see dashed line circle in the immunofluorescent images). Moreover, when we cultured the cells on the polystyrene dish supplemented with the conditioned medium collected from 5 d cultured cells on the copolymer substrates, the metabolic activity per cell was relatively higher than that of the cells with conditioned medium from the polystyrene dish (Figure 6f). However, this conditioned medium treatment experiment result did positively correlate with the increasing bulk stress relaxation time of the copolymer substrates. The latter two results suggest that copolymer substrates promoted parts of the breast cancer cell population to enter the senescence state after 5 d of cell culturing.

## 3. Discussion

The recent development of senolytic therapy as an adjuvant-tumor therapy to target senescent cells that enter the cell cycle arrest state signifies the role of cell cycle arrest fate in cancer. In this study, we proposed material-induced senescence as a trigger to induce cellular senescence in breast cancer cells, which relies on the generation of oxidative stress. Firstly, the cellular fate was initiated by the mechano-sensing of the surrounding matrix, followed by the force transduction occurring in the range of seconds timescale. The lifetime of the transient clutch formation between the cell adhesion receptor and its ligand determines cell adhesion to the matrix [42]. For example, the lifetime of clutch binding between LFA-1 (integrin $\alpha_{L}\beta_{2}$) and its ligand (ICAM-1) was a few seconds (1.80–10 s) in lymphocytes [43]. According to the molecular clutch theory, minimal cell spreading is expected to be observed when the relaxation time of the underlying substrate is less than the clutch binding timescale. This theory can be used to explain the absence of cell spreading (Figure 2a,b), followed by low paxillin expression (Figure 3a,b and Figure S17), causing the subsequent minimal actin stress fiber formation (Figure S15) on our viscoelastic liquid matrix with bulk relaxation time in the range of milliseconds. Instead, the MCF7 cells formed compact multicellular aggregates on the copolymer substrates (Figure 2a,b). The cell mechanosensing frequency was 1 Hz, based on the 1 µm/s actomyosin contraction rate and ~1 μm length of filopodia exerted specifically by the MCF7 cells [28,29]. In our viscoelastic liquid matrix, although the G′ also increased slightly when we altered the molecular weight of the copolymer, it only comprised 10% of the complex modulus at 1 Hz. The contradictory results obtained using our viscoelastic liquid matrix compared to the other studies using the hydrogel system are because the cells may not form a dense integrin clustering, as indicated by lower paxillin recruitment (Figure 3a,b and Figure S17). In other studies, investigating the cell culture on the liquid-liquid interface, the cultured mesenchymal stem cells could completely spread due to the spontaneously–formed protein film at the interface [44]. The fluorocarbon liquid, frequently used as the liquid-liquid interface for cell culture, has a polar group that can bind to the amino group of the protein, thus favoring the rigid protein film formation [45]. In our viscoelastic liquid substrate, regardless of the bulk relaxation time, the amount of adsorbed fibronectin was 25% lower than in the polystyrene dish (control), suggesting that the adsorbed fibronectin was in globular shape (folded state). This result agrees with the finding of another study using a soft gel with G′ of 144 Pa, in which the cells grown on the soft substrates were deficient in integrin–fibronectin bond strength and thus could not promote the fibrillogenesis of fibronectin required for the focal adhesion formation [38].

The cell-cell interaction is required to direct the cell cycle arrest of the breast cancer cells on the copolymer substrates because ROCK expression inhibition diminishes the expression of SA-β-Gal (Figure 4c). ROCK activates the tight junction proteins, such as claudins and occludin, in epithelial cells [46]. Although the tight junction proteins are disrupted in the senescent endothelial cell [47], they may not always occur in the context of epithelial cells. In addition, the senescent epithelial cells in the cell spheroid (3D culture) maintain the cell-cell interaction and do not gain the enlarged cell morphology commonly existing in a single cell state, as observed in a 2D culture [48]. Therefore, in this study, the senescence state of epithelial breast cancer cells may also be maintained by the presence of intact, tight junction proteins. In this case, the viscoelastic liquid nature of the substrates promoted the rapid movement of the cells from the first 24 h after the cell were seeded onto the substrates, leading to the coalescence of cells (Supplementary Video S1–S3). Depending on the bulk stress relaxation time of the copolymer substrates, the cells may remodel the fibronectin conformation to form the integrin clustering and eventually decrease their movement. On the polystyrene dish, the cells did not move across the substrate because the fibronectin conformation was in the unfolded state. Therefore, they can spread instantly and perform cell division (Supplementary Video S4). In our experiment, the range of the substrate bulk stress relaxation time was sufficient to induce the maturation of multicellular aggregates, as shown by their compact structures (Figure 2a). Therefore, the cell cycle arrest of the breast cancer cells on the copolymer substrates was initiated by the maturation of the multicellular aggregates formed by the cell-cell interaction.

The p38/MAPK phosphorylation indicated that the mechanism of the viscoelastic liquid-induced cell cycle arrest by tuning the substrate bulk stress relaxation time was mediated by oxidative stress (Figure 5a). However, the amount of ROS generated from the copolymer substrate-induced multicellular aggregates was similar to that from the ultralow adhesive surface-induced multicellular aggregates. The ultralow adhesive surface induced the formation of multicellular aggregates with a bigger size and higher compactness (Figure S11). This result confirms that the interplay of the bulk relaxation time and the viscoelastic liquid nature of the matrix can induce the confinement-like environment as in a compact 3D cell spheroid regardless of the cells assembling multicellular aggregates and their semi-3D structure. Moreover, the bulk stress relaxation of the viscoelastic substrate affected the size distribution of the formed multicellular aggregates (Figure 2e), in which the rapidly relaxed substrate generated a higher amount of small-sized multicellular aggregates (<80 µm). This result agrees with the common knowledge that the cell spheroid/multicellular aggregates with larger sizes produce a higher amount of ROS [49].

Dormancy corresponds to reversible cell cycle arrest. Nevertheless, it can seldom correspond to an irreversible cell cycle arrest or senescence state. Although the breast cancer cells express the senescence-associated lysosomal marker, SA-β-Gal, the expression of cell cycle arrest-related proteins (p27/Kip1, p53, and p21) were lower than that of their control counterparts (Figure 6a–d, Figure S13). In another study, an alginate hydrogel with a controlled stress relaxation time of ~1 s enables the reversible G1 phase cell cycle arrest of invasive breast cancer cells (MDA-MB-231) [20]. Our copolymer substrates also promote most breast cancer cell populations into the reversible S phase cell cycle arrest. However, we also demonstrate that tuning the stress relaxation of a viscoelastic substrate can control the deepening of dormancy into the senescence state, as shown by the Ki67 protein depletion in the polystyrene dish-replated cells (Figure 6e). Senescent cells exhibit a pro-tumorigenic effect on the neighboring cells by secreting the senescent-associated secretory phenotype [50]. This report is consistent with our results that the metabolic activity of breast cancer cells on the polystyrene dish supplemented with the conditioned medium of dormant and senescent cells on the copolymer substrates was 30% higher than that of the control substrate conditioned medium (Figure 6f).

Using the viscoelastic liquid substrate to investigate the matrix viscous component-induced dormancy of breast cancer cells is advantageous in generating viable multicellular aggregates. Dormant cells are viable cells with low metabolic activity and ceased proliferation ability [51,52]. The ultralow adhesive cell surface can promote the formation of compact 3-D multicellular aggregates, nevertheless with a necrotic core. Moreover, the disseminated breast cancer cells usually found at the endosteal lining of the bone marrow need to adhere to the niche via integrin-mediated adhesion before becoming dormant [53]. The integrin clustering of those dormant breast cancer cells is lower than their normal counterparts [54]. The viscoelastic liquid matrix enabled the surface anchorage of the multicellular aggregates, and the cells within the multicellular aggregates remained viable over 5 d cell culture, distinguishing it from the ultralow adhesive cell culture surface. The viscoelastic liquid matrix in this study provides a low adhesion surface based on the rheological factor; meanwhile, the ultralow adhesive surface provides a low adhesion surface through surface chemical modification. Although the ultralow adhesive surface can induce the formation of multicellular aggregates, these aggregates did not anchor to the surface and form the necrotic core during 5 d cell culture; thus, it does not represent the dormancy state properly. In contrast, the cell in the dormancy state only ceases its proliferation while maintaining its viability [52].

Numerous studies have been confirming the role of the bone marrow niche in inducing the dormancy of the disseminated breast cancer cells via biochemical factors secreted by the bone stromal cells [55]. Bone marrow, a fluid-type body tissue, is dominated by the yellow marrow (adipocyte) as we age [56], implying the potential change in its mechanics over a long period of the human lifespan. Several studies have investigated the mechanism of cancer cell dormancy mediated via altered matrix viscoelasticity using a hydrogel platform [10,20,27,57,58]. They reported how stress relaxation drives the spreading of human mesenchymal stem cells in a matrix with low elastic modulus or promotes breast cancer cell growth arrest; within the time range of 80–10,000 s [19,20]. To the best of our knowledge, our study is the first to confirm that breast cancer cells (including the invasive type, MDA-MB-231 (Figure S14)) can sense the narrower range of substrate relaxation time (in milliseconds), 1000 times lower than those in previously reported studies. At this range of substrate relaxation time (milliseconds), breast cancer cell dormancy was induced in a contrasting manner to reports on a solid substrate of alginate-based hydrogel despite the different cell lines being used [20]. Our viscoelastic liquid matrix also induces different cell behavior compared with the liquid-liquid interface. At the liquid-liquid interface of ionic liquid and cell culture medium, the invasive breast cancer cell line can completely spread and proliferate in a certain concentration range of imidazolium ionic liquid [59]. Our findings also provide a new perspective regarding the dormancy mechanism of breast cancer cells concerning the stress relaxation time of the underlying substrate and the possibility of material-induced senescence induction of breast cancer cells.

## 4. Materials and Methods

### 4.1. Substrate Preparation

A four-branched copolymer, P(CL-*co*-DLLA), was synthesized by ring-opening copolymerization of pentaerythritol using a tin octanoate catalyst, as described in our previous studies [60,61]. The chemical structures were confirmed by proton nuclear magnetic resonance (^1^H NMR) spectroscopy (Jeol, Tokyo, Japan). The molecular weights were estimated by gel permeation chromatography (GPC, JASCO International, Tokyo, Japan) using polystyrenes (Tosoh Corporation, Tokyo, Japan) as the calibration standard and tetrahydrofuran (THF, Fujifilm Wako Pure Chemical Corporation, Osaka, Japan) as the elution solvent. P(CL-co-DLLA) with molecular weights of 11.7 and 7.2 k were dissolved in toluene at 20 and 50 wt%. The polymer solution was coated on ethanol-washed and ultraviolet (UV)-sterilized 22 mm or 15 mm glass coverslips (Matsunami Glass, Osaka, Japan) at 3000 rpm for 2 min by spin coating (Active Spin Coater, Axel, Tokyo, Japan) and subjected to solvent drying at 60 °C for 24 h.

### 4.2. The Wettability and Surface Morphology of the Substrates

The wettability of fluidic substrates was determined using a contact angle meter (Drop Master, Saitama, Japan). The water contact angle values were taken at three different positions for each glass coverslip at room temperature (25 °C). The surface morphology of the fluidic substrates was imaged by using scanning probe microscopy (SPM) (Bruker Co., Minneapolis, MN, USA) operating in tapping mode in the air. Silicon-nitride cantilever (Budget Sensors, Sofia, Bulgaria) with force constant of 3 N/m and resonance frequency of 75 kHz was used.

### 4.3. Protein Adsorption of the Substrates

The fluidic substrates were immersed in fibronectin (Sigma-Aldrich, St. Louis, MO, USA) solution (10 µg/mL, 1 h, 37 °C). After adsorption, substrates were washed with phosphate-buffered saline (PBS, Nacalai Tesque, Kyoto, Japan) three times to remove the unabsorbed protein. The concentration of adsorbed fibronectin was quantified by using the micro BCA protein assay kit (Thermo Fisher Scientific, Waltham, MA, USA) according to the manufacturer’s instructions. The absorbance was measured using a microplate reader (Tecan, Männedorf, Switzerland) at 562 nm. The experiment was performed in triplicate.

### 4.4. Bulk Mechanical Characterization of the Substrates

The rheological measurement of fluidic material was conducted using an MCR 301 rheometer (Anton Paar, Ostfildern, Germany). Fluidic substrates were placed between two parallel PP-10 plates with a gap distance of 0.2 mm. Viscoelasticity spectra (storage modulus (G′) and loss modulus (G′′) were obtained under frequency sweep mode with fixed strain concentration (γ = 1%) from 0.1 to 500/s at constant temperature (37 °C). The stress relaxation of the fluidic material was measured using the same rheometer at the constant strain concentration of 10%, and the relaxation modulus was monitored for 1000 s. The stress relaxation time was calculated by fitting the nanoindentation data to the standard linear solid model using the MATLAB curve fitting toolbox:

$$\varepsilon \left(t\right)=(\sigma_{0}/E_{1})\left(1-\frac{E_{2}}{E_{1}+E_{2}}\right)e^{-t/\tau }$$

where *σ*_0_ is initial stress, *E*_1_ & *E*_2_ are viscoelastic parameters, and *τ* is the relaxation time of the material.

### 4.5. Fluorescence Recovery after Photobleaching (FRAP) Assay

The substrates for FRAP experiments were prepared by the spin-coating of 50 wt% of polymer solution in toluene (Fujifilm Wako Pure Chemical Corporation) containing 0.01 wt% of Nile red (Tokyo Chemical Industry, Tokyo, Japan) at 3000 rpm for 100 s. FRAP experiments were performed on a Leica SP5 confocal microscope (Leica, Wetzlar, Germany). Imaging was performed using a 20× objective at 2.5× digital zoom at 700 Hz. The confocal pinhole was set at 1.79 Airy units. The imaging window contained a circular bleach ROI of 3 μm diameter. FRAP conditions were optimized for each molecule understudy. Substrates were imaged using an Argon laser (488 nm). The setup was to take five pre bleach images, bleach with only one image, and follow the recovery for 50 images. All FRAP experiments were conducted at room temperature (25 °C) in the air. The data were corrected and analyzed to obtain the half-time recovery and diffusion coefficient, as described previously [62].

### 4.6. Surface Mechanical Properties of the Fluidic Substrates

Nanoindentation experiments were performed using a Hysitron Triboindenter 950 (Hysitron, Minneapolis, MN, USA) with a Berkovich indenter. In order to obtain the force relaxation of the substrates; a constant displacement mode was used. The displacement was maintained at 300 nm, and the holding time was set for 60 s for all samples. The stress relaxation time was calculated by fitting the nanoindentation data into the Maxwell model representing the linear viscoelastic material using the MATLAB curve fitting toolbox:

$$\sigma =\sigma_{0}e^{-t/\tau_{0}}$$

where σ_0_ is the initial stress and *τ*_0_ is the relaxation time of the material.

### 4.7. Evaluation of the Fluidic Substrate Thickness

The thickness of each substrate was measured using Leica SP5 confocal laser scanning microscope (Leica). The substrates were prepared by spin-coating of Nile red containing polymer solution (same as FRAP samples) at 3000 rpm for 100 s. The z-stack images of each substrate were acquired at every 0.5 µm image slice. The z-stack images were then 3D projected at a 90° angle, and the distance across the image was measured as the substrate thickness.

### 4.8. Cell Culture

The MCF-7 cells (RIKEN BioResource Research Center, Tokyo, Japan) were grown in Eagle’s minimum essential medium (Sigma-Aldrich) supplemented with 10% heat-inactivated fetal bovine serum (FBS; Sigma-Aldrich), 1% L-glutamine (Sigma-Aldrich), and 1% penicillin-streptomycin (Sigma-Aldrich). Cells were maintained at 37 °C under a humidified atmosphere of 5% CO_2_. Subculturing was performed every three days with 0.25% trypsin-EDTA (Nacalai Tesque) until use. The prepared fluidic substrates were sterilized using ethylene oxide gas (Nippon Ekitan Corporation, Fukuoka, Japan) at 55 °C for 18 h and coated with 10 µg/mL fibronectin for 1 h at 37 °C before cell culture. Finally, the cells were cultured on the substrate for 1–5 d.

### 4.9. Cell Metabolic Activity Assay

The cells were seeded at 2 × 10^4^ cells/well on the fluidic substrates set on a 24-wells plate using a UV-cured glue (1–5 d). The cells cultured directly into the well served as a control. The metabolic activity of the cells was evaluated by using the CCK-8 assay kit (Dojindo Molecular Technologies, Rockville, MD, USA) according to the manufacturer’s protocol. The cells were incubated with the CCK-8 reagent for 2 h. The absorbance of the samples was measured at 450 nm using a microplate reader (Tecan) and then normalized to the control sample.

### 4.10. Cell Proliferation Assay

The cells cultured on each substrate were washed with PBS three times and lysed with lysis buffer for 15 min at room temperature (25 °C). The Quant-iT PicoGreen dsDNA Assay Kit (Invitrogen, Waltham, MA, USA) was used to quantify the DNA content of the cell lysates. The cell numbers were calculated based on the cell DNA using a calibration curve of total cell DNA versus known numbers of cells. PicoGreen (excitation λ: 480 nm/emission λ: 520 nm) fluorescence intensity was measured using an Infinite^®^ 200 PRO fluorescence plate reader (Tecan).

### 4.11. Cell Death Assay

The percentage of dead cells was determined by cytotoxicity LDH assay kit (Dojindo Molecular Technologies) according to the manufacturer’s instructions by using the supernatant-based assay. In addition, the cell death marker expression of the cells cultured on each substrate was assessed using Annexin V (apoptosis) and PI (necrosis) staining (Apoptotic/Necrotic/Healthy Cells Detection Kit, PromoCell GmbH, Heidelberg, Germany). Cells were trypsinized, and the number was adjusted to approximately 10^6^ cells for each sample, followed by incubation with those markers in the dark for 30 min at room temperature (25 °C). The cells were flown into a flow cytometer (Sony SH800S; Sony Biotechnology, San Jose, CA, USA).

### 4.12. Cell Cycle Assay

Cells were seeded on the substrates and coverslips 10^5^ cells/well. After 5 d, the cells were collected by trypsinization, and the cells number was adjusted to approximately 10^6^ cells for each sample by quantifying the Trypan Blue (Bio-Rad Laboratories, Hercules, CA, United States)-stained cells using an automated cell counter (TC20, Bio-Rad Laboratories). These samples were then fixed with chilled 70% ethanol for 30 min at −20 °C and washed three times with PBS. The cells were stained with 500 µL FxCycle™ PI/RNase staining solution (Invitrogen) for 30 min in the dark at room temperature (25 °C), followed by the cell cycle analysis using a flow cytometer (Sony SH800S). The data analysis (gating) of each cell cycle phase was performed using the ModFit LT™ software.

### 4.13. Immunofluorescence Analysis

Cells were seeded on the fluidic substrates and coverslip glass at 3 × 10^4^ cells/cm^2^ and incubated for the required periods. The cells were fixed in 4% paraformaldehyde (Fujifilm Wako Pure Chemical Corporation) and blocked with 2% bovine serum albumin (BSA; Sigma-Aldrich) (in PBS) for 1 h. The paxillin, and p-p38/MAPK were stained independently with anti-paxillin antibody and anti-p-p38/MAPK antibody respectively (Santacruz Biotechnology, Dallas, TX, USA), and the corresponding secondary antibody conjugated with Alexa Fluor^®^ 488 fluorescent dye (Invitrogen) for 1.5 h each. F-actin and nuclei were counterstained with tetra-methyl rhodamine B isothiocyanate-conjugated phalloidin (Sigma-Aldrich) and DAPI (Sigma-Aldrich), respectively. The images were taken using a confocal microscope (Zeiss LSM980, Carl Zeiss AG, Oberkochen, Germany) and processed with Zen Blue System software.

### 4.14. Western Blot

The cells were lysed with RIPA buffer (Sigma-Aldrich) for 30 min at 4 °C and centrifuged at 9000 rpm for 20 min at 4 °C. The protein concentration was measured using the micro-BCA assay kit (Invitrogen). Next, the protein samples were denatured at 95 °C for 5 min and were loaded at 20 µg per well along with 5 µL of prestained protein marker (Nippon Gene, Tokyo, Japan) into 10–20% or 15% mini polyacrylamide gel (Atto, Tokyo, Japan) in a reduced condition. The gel was then transferred onto a PVDF membrane (Invitrogen) using a semi-dry blotting method. The membranes were blocked in 3% skim milk (Sigma-Aldrich) before incubation with the following antibodies: anti–p27/Kip1 (Santa Cruz Biotechnology), anti–p53 (Santacruz Biotechnology), anti–p21 (Santacruz Biotechnology), anti–HIF1α antibody (R&D Systems, Minneapolis, MN, USA), anti–Ki67 antibody (R&D Systems), and an anti–GAPDH (R&D Systems). The membranes were then conjugated with anti–HRP secondary antibodies. Before detecting protein bands using a luminescent gel imager (LumiCube; Liponics, Tokyo, Japan), the membrane was incubated with ECL western blotting detection reagents (Millipore Sigma, Burlington, MA, United States) for 5 min in the dark. Finally, the protein bands were quantified using ImageJ software by normalizing the band intensity of each protein marker to the GAPDH protein band as the control.

### 4.15. Senescent-Associated-β-Galactosidase Staining and Quantification

Cells were seeded 2.5 × 10^4^ cells/well on fluidic substrates and glass coverslips in 24-well plate inserts and cultured for different time points (1–5 d). Then, the cells were fixed and stained for senescence-associated beta-galactosidase (SA-β-Gal) at pH 6.0 using Senescence Cells Histochemical Staining Kit (Sigma-Aldrich) according to the manufacturer’s protocol. SA-β-Gal-stained cell images were observed under a phase-contrast microscope (ECLIPSE Ti2; Nikon, Tokyo, Japan). The senescent cells were quantified using the cellular senescence plate assay kit SPIDER-β-Gal (Dojindo Molecular Technologies) according to the manufacturer’s protocol (excitation λ: 535 nm/emission λ: 580 nm) after cell lysis. SPIDER-β-Gal fluorescence intensity was measured using a fluorescence plate reader (Tecan).

### 4.16. Inhibition Study

For pharmacological inhibition, 20 µM Y27632 (MedChemExpress, Monmouth Junction, NJ, USA) and 20 µM mitoquinone mesylate (Selleck Chemicals, Houston, TX, USA) were used, and cells were treated with each drug for 24 h. The effect of the inhibitor treatment on the senescence induction of MCF-7 cells was confirmed by examining the expression of SA-β-Gal and SPIDERβ-Gal using the previously mentioned kits.

### 4.17. Cell Replating Experiment

First, 3 × 10^4^ cells/well were seeded onto each substrate, and the cells were cultured for 5 d. Cells were trypsinized, and the number was adjusted to 3 × 10^4^ cells/well for subsequent seeding into a normal polystyrene tissue culture plastic dish for 2 d of continuous culture. At the end of the cell culture, the cells were fixed with 4% PFA (FUJIFILM Wako Pure Chemical Corporation), and immunofluorescence staining of anti-Ki67 and DAPI was performed using the similar procedure mentioned above.

### 4.18. Statistical Analysis

All results are represented as the mean obtained from three independent experiments. The degree of significance of each data for P(CL-*co*-DLLA) substrates with different molecular weights compared with tissue culture plastic dish was analyzed by Student’s *t*-test, where *p* < 0.05 was considered as statistically significant.

## 5. Conclusions

The viscoelastic liquid substrate was developed to study the dormancy induction of breast cancer cells in the bone marrow matrix. In this system, a threshold of bulk stress relaxation time that induce the highest number of cells to undergo senescence may exist between 80 and 210 ms. The cell cycle arrest of the breast cancer cells on the copolymer substrates is initiated by the formation of mature multicellular aggregates followed by ROS generation, leading to the complete arrest at the S phase. This study highlights the possibility of tuning the bulk stress relaxation of the viscoelastic liquid substrates to direct the dormancy of the breast cancer cells and deepen the dormancy state into the senescence state. Altogether, this study supports the strategy of inhibiting the integrin-mediated cell adhesion for inhibiting dormancy of the disseminated breast cancer cell in a viscous matrix, such as the bone marrow.

## Data Availability

The raw/processed data required to reproduce these findings are available upon request.

## Supplementary Materials

The following supporting information can be downloaded at: https://www.mdpi.com/article/10.3390/ijms232314637/s1.
