# Supplementary material for: Viscoelastic Liquid Matrix with Faster Bulk Relaxation Time Reinforces the Cell Cycle Arrest Induction of the Breast Cancer Cells via Oxidative Stress
Source: Int J Mol Sci. 2022 Nov 24;23(23):14637. doi: 10.3390/ijms232314637 (PMC9736955; doi:10.3390/ijms232314637)
Supplement: Supplementary file 1 [file ijms-23-14637-s001.zip › ijms-2005462-supplementary.pdf]

Supplementary Information

Viscoelastic liquid matrix with faster bulk relaxation time reinforces the cell cycle arrest induction of the breast cancer cells via oxidative stress

Mazaya Najmina[1,2], Mitsuhiro Ebara[1,2,4], Takahito Ohmura[3], Koichiro Uto[1]*

[1] Research Center for Functional Materials, National Institute for Materials Science, 1-1 Namiki, Tsukuba, Ibaraki, 305-0044, Japan

[2] Graduate School of Science and Engineering, University of Tsukuba, 1-1 Tennodai, Tsukuba, Ibaraki, 305-8577, Japan

[3] Research Center for Structural Materials, National Institute for Materials Science, 1-2-1 Sengen, Tsukuba, Ibaraki, 305-0047, Japan

[4] Graduate School of Industrial Science, Tokyo University of Science, 1 -3 Kagurazaka, Shinjuku, Tokyo, 162-8601, Japan

**Corresponding author: UTO.Koichiro@nims.go.jp*

Table S1: The composition and molecular characteristics of polymers.

Type	CL/DLLA in feed	CL/DLLA in copolymer[a]	Copolymer[b]		
			M_w, g/mol	M_n, g/mol	PDI
4b-130	1.5	1.6	35k	19.3k	1.9
4b-160		1.6	41k	25.k	1.6
4b-250		1.6	63k	38.5k	1.7

[a] determined by ^{1}H-NMR (Solvent: $CDCl_3$) [b] estimated by GPC (solvent: DMF, standard: polyethylene oxide

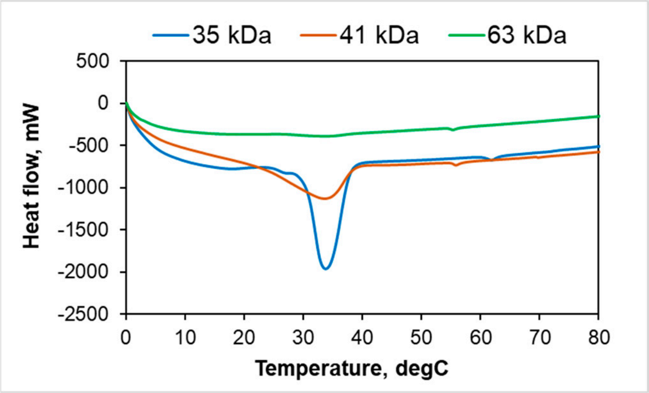

Figure S1: The second scan differential scanning calorimetry (DSC) thermogram of P(CL-*co*-DLLA).

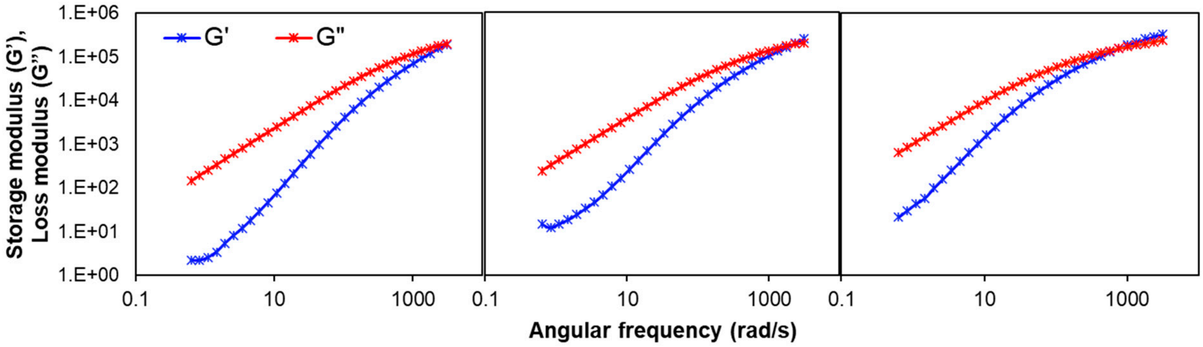

Figure S2: Storage modulus (G') and loss modulus (G") of P(CL-*co*-DLLA) with various molecular weight [35 kDa (left), 41 kDa (middle), and 63 kDa (right)].

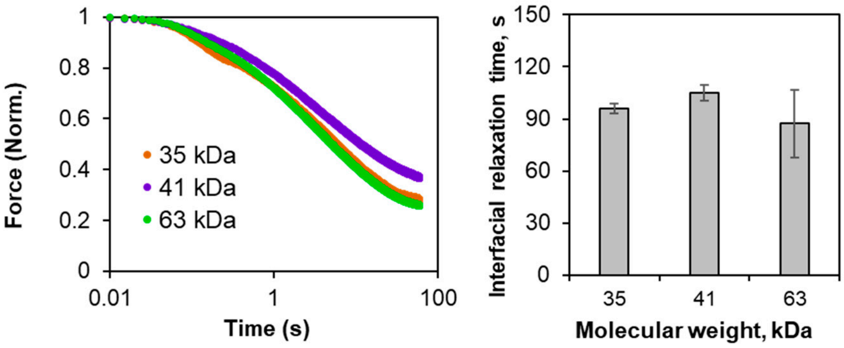

Figure S3: The force-time curve (normalized to each individual maximum force) measured by nanoindenter (at 300 nm from surface) and the interfacial relaxation time obtained by curve fitting of the force-time curve to the Maxwell model.

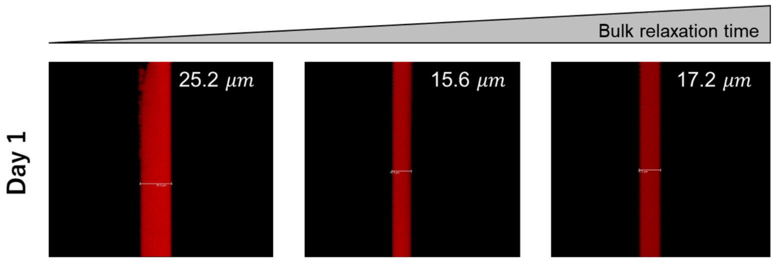

Figure S4: The thickness of polymer substrates indicated by the z-axis view red fluorescence after 1 day immersion in cell culture medium

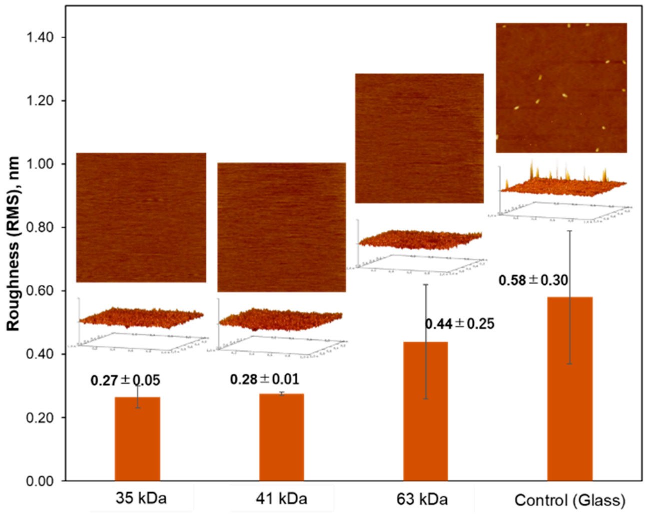

Figure S5: The RMS roughness of P(CL-*co*-DLLA) substrate obtained by AFM.

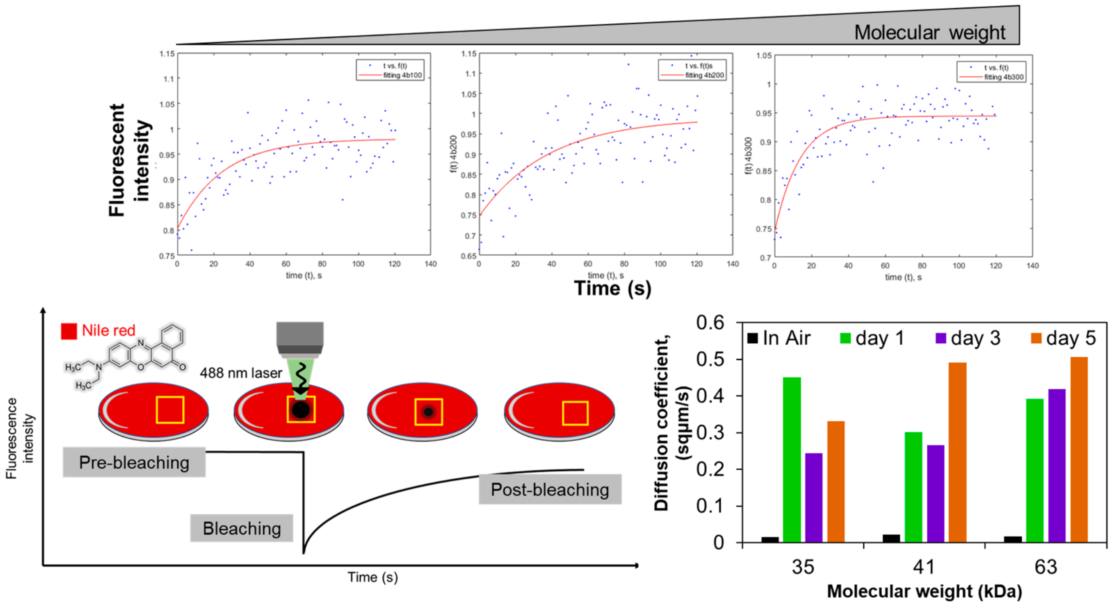

Figure S6: The mobility P(CL-*co*-DLLA) spin-coated substrate probed by nile-red measured by fluorescence recovery after photobleaching (FRAP) in air and in PBS (day

1, day 3, day 5).

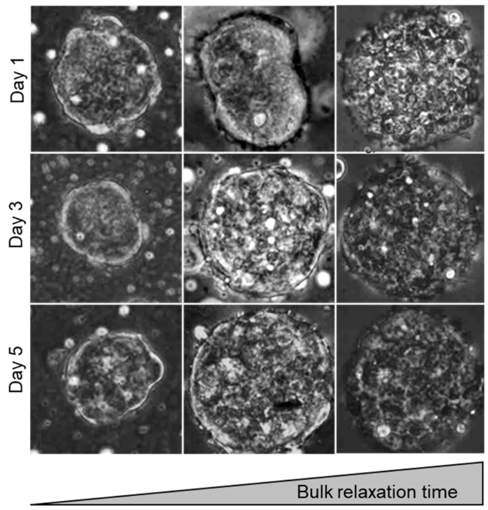

Figure S7: The morphology of the multicellular aggregates over 5 days cell culture.

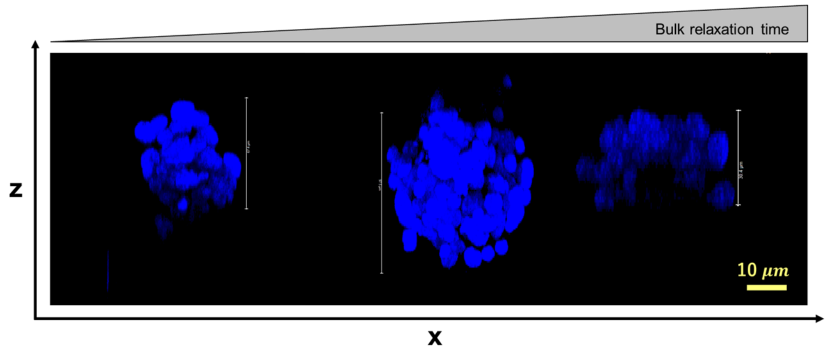

Figure S8: The structure of the multicellular aggregates formed on each polymer substrates viewed from the z-axis of DAPI-stained cells using a confocal microscopy at day 3 of the cell culture.

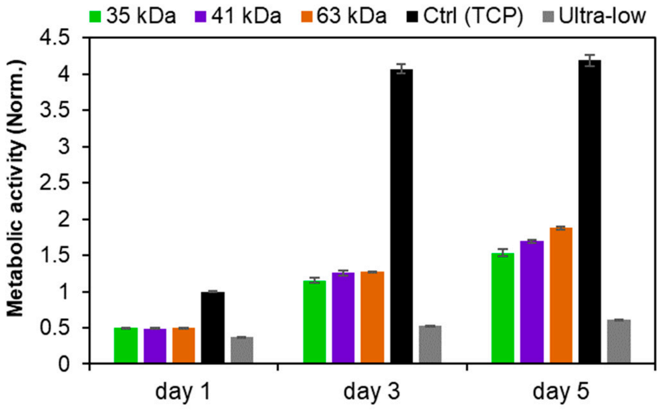

Figure S9: The metabolic activity (NAD^{+}→NADH) of breast cancer cells cultured on various substrates (viscoelastic liquid substrates, and ultralow adhesive surface, polystyrene) from day 1 to day 5 of cell culture.

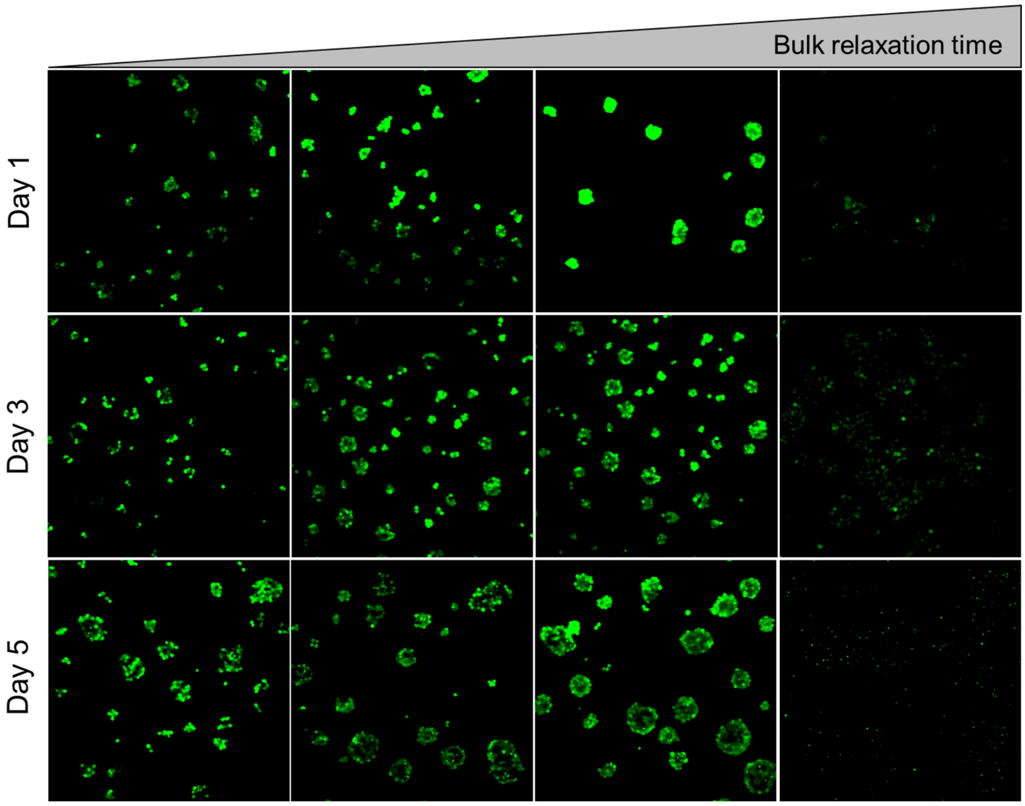

Figure S10: The DCFDA expression of MCF7 cells on substrates with various bulk relaxation time indicating the intracellular ROS generation of cells.

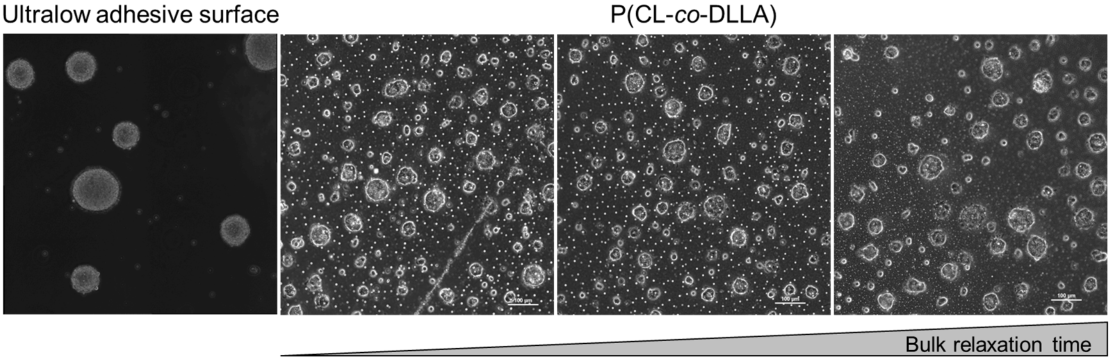

Figure S11: The morphology of the multicellular aggregates formed on the ultralow adhesive surface

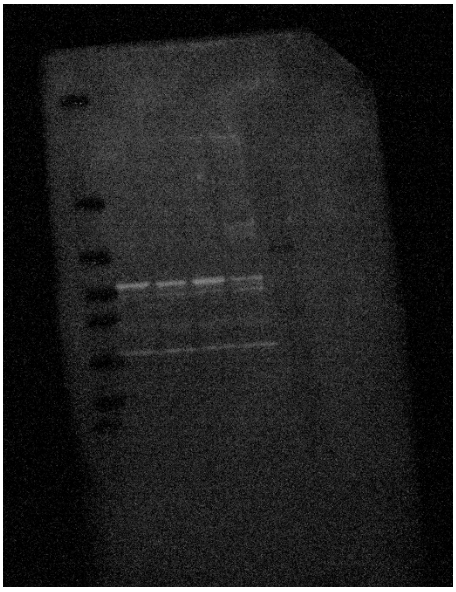

Figure S12: The full western blot gel image of HIF1A in Figure 5e.

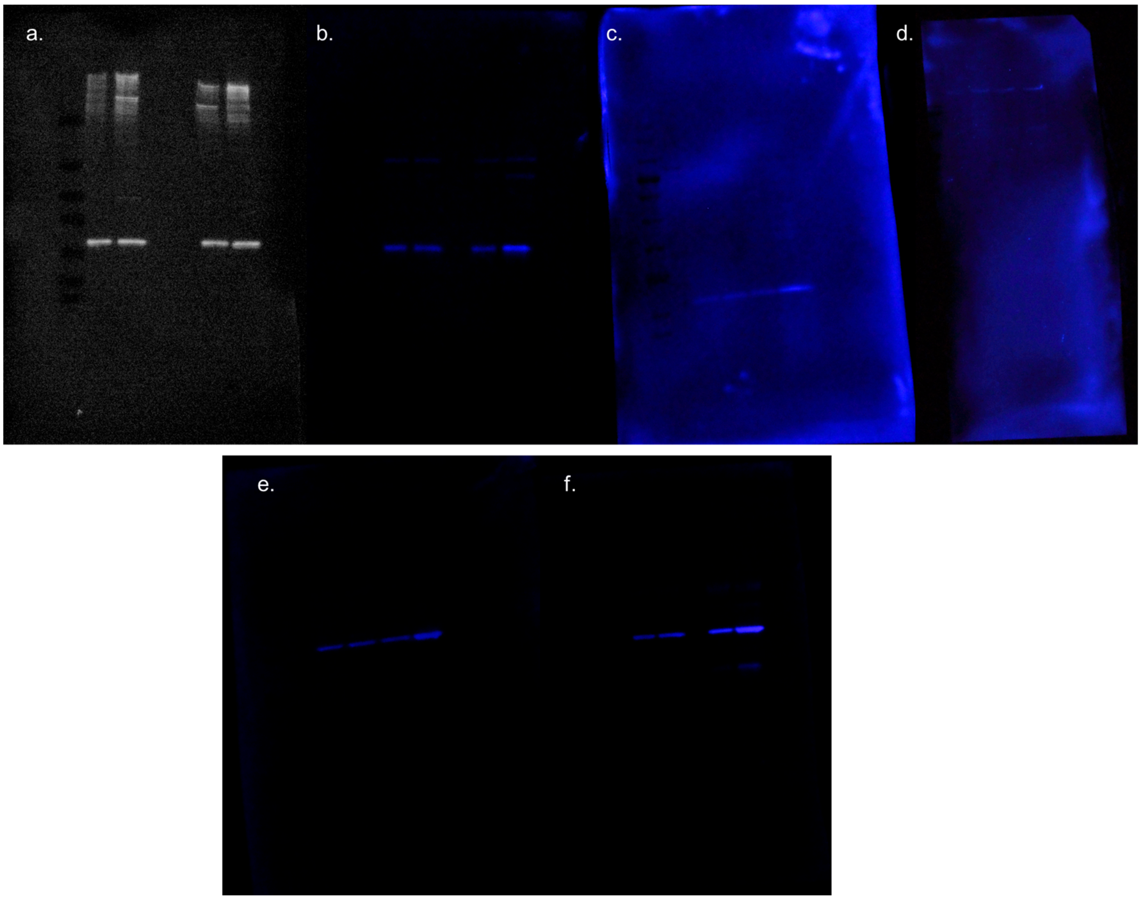

Figure S13: The full western blot gel image of (a.) Ki67 (Figure 6a); (b.) p27/Kip1 (Figure 6b); (c.) p21 (Figure 6c); (d.) p53 (Figure 6d); (e.) GAPDH for p21 and p53; (f.) GAPDH for p27/Kip1.

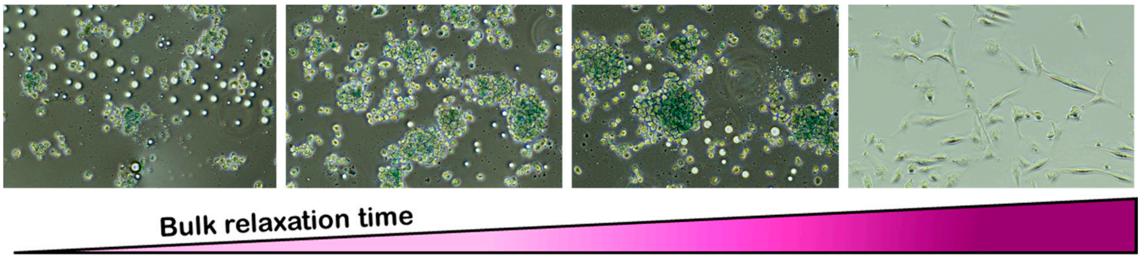

Figure S14: The SA-β-Gal staining of MDAMB231 cell line (3 x 10^4 cells/ well) cultured on various substrates with various bulk relaxation time for 3 days (the green colored part in each image indicates positive expression of SA-β-Gal) (10x magnification).

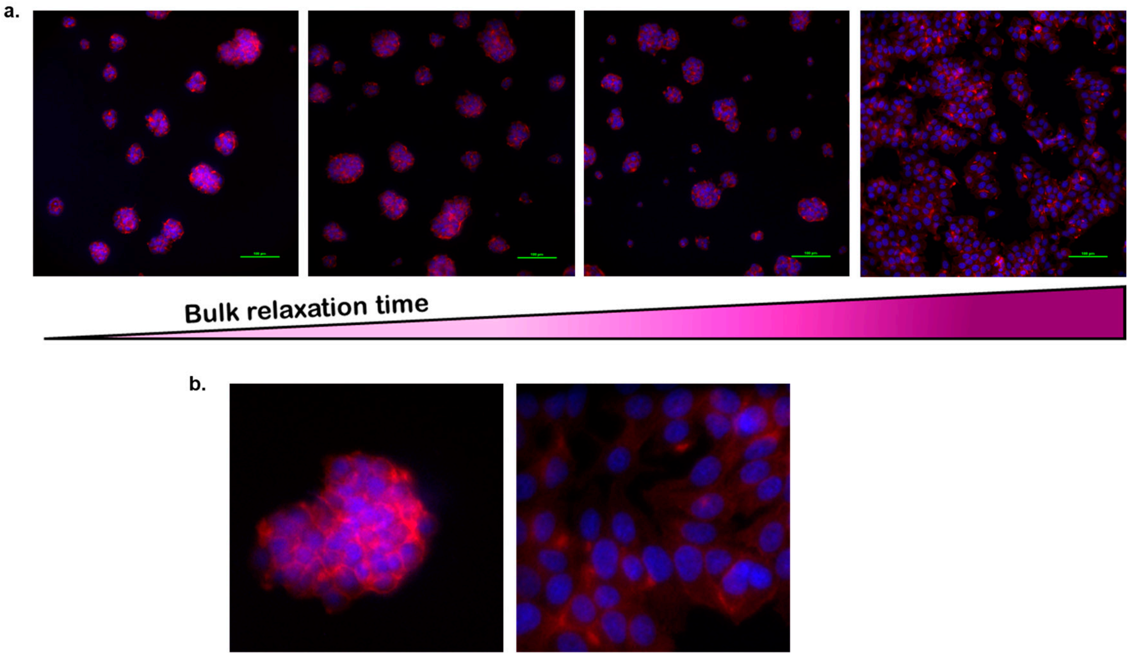

Figure S15: (a.) The merged actin (red) and DAPI (blue) staining images of MCF7 cells on the various substrates with various bulk relaxation time (10x magnification); (b.) The actin staining images of MCF7 on the copolymer substrate (left) and on the TCP (right).

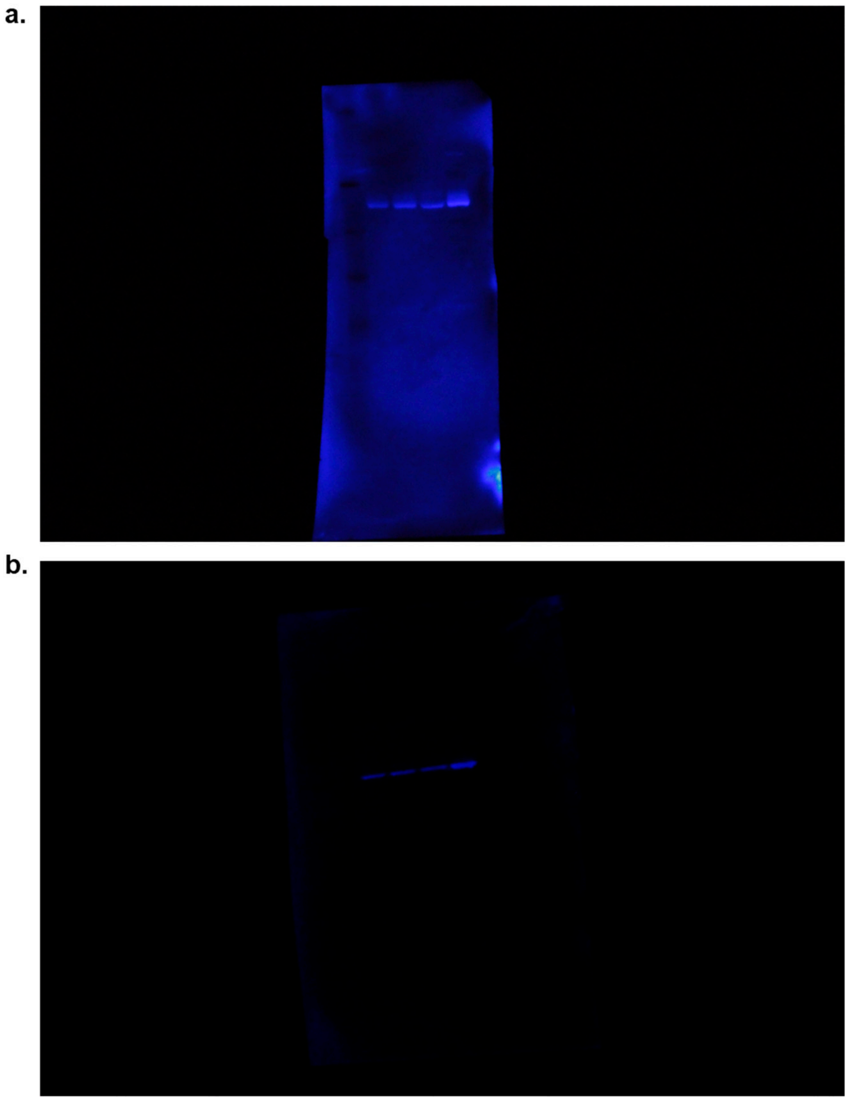

Figure S16: The paxillin expression of MCF7 cells on the various substrates with various bulk relaxation time (Left to right: MCF7 cells on 80 ms, 290 ms, 210 ms, and 1e+9 ms substrate).

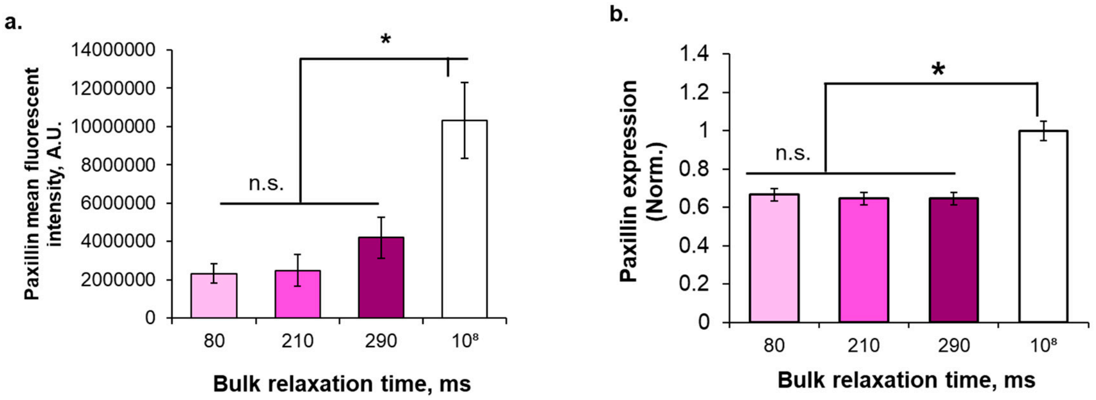

Figure S17: (a.) The quantified fluorescent intensity of Paxillin of MCF7 on various substrates with various bulk relaxation time (normalized to cell area) based on Figure 4a and (b.) The quantified western blot paxillin expression (Figure S16a) of MCF7 cells on the various substrates with various bulk relaxation time (Normalized to the GAPDH expression in Figure S16b).
